# Expectations of healthcare professionals of community-based telemedicine in emergency medical service

**DOI:** 10.1371/journal.pone.0310895

**Published:** 2024-09-19

**Authors:** Elisabeth Klager, Anna Teufel, Magdalena Eitenberger, Nils Bukowski, Josef Michael Lintschinger, Valerie Manschein, Philipp Metelka, Harald Willschke, Eva Schaden, Christoph Frimmel, Reinhold Renner, Christina Hafner

**Affiliations:** 1 Ludwig Boltzmann Institute Digital Health and Patient Safety, Vienna, Austria; 2 Department of Anaesthesia, Medical University of Vienna, General Intensive Care and Pain Medicine, Vienna, Austria; 3 Austrian Red Cross, State Association of Burgenland, Eisenstadt, Austria; San Giuseppe Hospital, ITALY

## Abstract

**Background:**

In times of demographic change and an immense shortage of qualified personnel in emergency medical services, telemedicine could offer more efficient solutions for better care. Given the community-based nature of emergency services, local communities play an important role. This study explored the expectations of healthcare professionals and volunteers for telemedicine tools in prehospital emergency medicine.

**Methods:**

This mixed-methods study was conducted in the rural region of Burgenland in Austria with stakeholders of the local emergency medical service in two focus groups (13 participants) and 99 quantitative questionnaires.

**Results:**

Combining quantitative and qualitative data, we found that a majority of respondents (almost 80%) already experienced basic telemedicine and consider it valuable. In particular, there is a strong expectation for diagnostic support and inquiries related to potential hospitalization. Findings from two focus groups emphasized the importance of cultivating an improved learning culture, developing a specific mindset, and refining soft skills. The optimal telemedicine solution includes a knowledgeable and experienced tele-emergency physician coupled with user-friendly technology.

**Conclusion:**

To be clear about the expectations of stakeholders, it is essential to involve all stakeholders right from the beginning. The solution should prioritize the integration of existing structures and be seamlessly incorporated into an evolving learning culture, while also fostering the necessary mindsets alongside educational aspects.

## Introduction

Over the past years, especially in rural areas, emergency medical services (EMS) have struggled with increasing numbers of deployments requiring input from emergency physicians (EPs). Additional challenges posed by sociodemographic change and increasing shortages of healthcare professionals (HCP) exacerbated the situation [1,2]. In Austria, the EMS is a combined system involving on-site emergency medical technicians (EMTs) and prehospital EPs who are dispatched separately according to the specific situation. Since most ground-based ambulance staff in rural regions of Austria are volunteer EMTs, local communities play a significant role in providing prehospital emergency care. Given the current challenges and technological advances, the opportunity for HCPs and volunteer EMTs to use telemedicine (defined by the WHO as “the use of digital technologies to overcome distance barriers in delivering health services”) is a highly relevant matter [3,4]. Rural areas in particular could benefit as time is a critical factor in EMS and it currently takes an average of up to fifteen minutes to reach the patient in certain rural areas in Austria and Germany [5]. This might have a negative impact on patients and is a burden on the entire system [6]. Also a recent study by Pietrantonio et al. (2023) reported that telemedicine enhances patient safety and provides rapid response in emergencies [7]. In another study from 2023, a team in Germany analysed whether the routine use of a tele-emergency medical service is as effective as a conventional physician-based service in terms of intervention-related adverse events. They found that in severe emergency cases, the tele-emergency medical service was indeed non-inferior to the conventional physician-based service regarding the occurrence of adverse events [8]. Despite the potential benefits, healthcare systems struggle with the implementation and dissemination of healthcare innovations. Countermeasures and strategies can be used to address this–from finding solid innovations to identifying and supporting “innovators” to empowering “early adopters” [9]. Still, even with several recent prehospital telehealth initiatives in various countries, a high proportion has failed to transition from pilot projects to routine use [10]. What the positive and still ongoing projects have been able to demonstrate, however, is that successfully implementing a tele-emergency system with tele-emergency physicians (TEPs) depends on addressing the needs and concerns of all relevant stakeholders. While user-centred innovation has been gaining ground in the consumer sector for decades, it has only recently found its way into the field of science and the health sector [11,12]. The resulting open innovation in science approach is a process that involves all stakeholders–experts, general public, and those directly affected such as patients–from the outset [12]. It thus seems essential that projects for a community-based emergency medical system involving HCPs and volunteer EMTs are embedded into the local structures and community to be successful. This should ensure that the solution will facilitate and improve workflows and enhance patient safety by enabling the best possible care.

The aim of this mixed-methods study was to involve relevant stakeholders of a rural region in Austria prior to the development and implementation of a telemedicine system for EMS and to record their expectations, needs and concerns.

## Methods and materials

This study was conceptualized as mixed-methods study using quantitative and qualitative data and triangulating it for a deeper understanding [13]. Burgenland in Austria was chosen as the ideal regional environment, as distances are particularly large due to the sparse population. In addition, the average age is relatively high, and the current system is dependent on volunteers, demonstrating a particular need for a new prehospital emergency care strategy. These characteristics are widespread in rural areas in Austria and Germany, so that reproducibility and a certain representativeness can be expected.

The study is embedded in a long-term project to co-design and implement telemedicine in EMS in rural Austria. The project uses the PODUCES frameworks by Leask and other methods to involve stakeholders in all phases from problem definition to implementation and evaluation [14,15]. We involved emergency system stakeholders (i.e., EPs, full-time and volunteer EMTs, call centre employees and government representatives), asking about their experiences, concerns, and ideas for creating a solution for telemedicine in EMS. This study was exempted from the approval requirement by the Chairman of the Ethics Committee for the Vienna Hospitals in Vinzenz Holding, as it is based on the voluntary participation of interested persons working in EMS and no personal data was collected. This exemption is in accordance with Austrian law and the General Data Protection Regulation of the European Union (GDPR).

### Patient and public involvement

Involvement of stakeholders is of high importance in this project including local politics and community based voluntary EMTs next to EPs. EMTs and EPs have been involved in the study as participants in the online survey and as focus groups members.

### Data collection and analysis of quantitative data

Quantitative data was collected via a survey following CHERRIES guidelines [16]. It targeted active EMS healthcare providers in Burgenland. Participants were informed about the survey’s duration (5–7 minutes), anonymity of data, and adherence to data protection regulations. Before starting the questionnaire, participants had to give their consent to take part in the online survey within the questionnaire. The online questionnaire (S1 File), developed by experts in EMS and open innovation, consisted of 32 items, including single choice, multiple choice, and open questions and was hosted by the Unipark platform. Responses were collected through the platform and pseudonymised data were stored on password protected computers at the Ludwig Boltzmann Institute Digital Health and Patient Safety. Access to the online survey was distributed via email, relevant newsletters, and internal information channels to 772 active EMS healthcare professionals in Burgenland between April 15 and June 15, 2022. Statistical analysis was performed with Prism 9.0 software (GraphPad, San Diego, CA, USA). Descriptive statistics were used to report the results of the survey. Furthermore, two subgroup analyses have been performed using an ordinary least squares linear regression model on age, sex and medical education to predict the acceptance of telemedicine support. Subjects with missing data were dropped, leaving 86 subjects for regression analysis.

### Data collection and analysis of qualitative data

For the qualitative data COREQ was used [17]. Two online focus groups were conducted via Zoom by a sociologist of the research team, each lasting 120 minutes. A predetermined self-generated question guide (S2 File) was used to explore expectations and concerns related to telemedicine, while allowing flexibility for other relevant topics. Participants (selected and invited by the research team) were assured that data would be processed anonymously. Before starting the focus group all participants gave individual oral consent to participate in the study and to be recorded. The discussion of the focus groups was recorded and transcribed. The first step of the thematic analysis was to familiarise ourselves with the data to obtain a general overview [18]. A total number of 23 codes were identified and selected based on relevance to the research question. Where necessary, new codes and subcodes were defined during the iterative process. The coding process was conducted by two researchers using printed versions of the transcribed focus groups. Selected quotes were directly highlighted to enhance and support the immediacy of the findings [17].

## Results

### Results of the quantitative online survey

99 healthcare professionals working in ground-based prehospital EMS in the rural area of Burgenland (59% EMT-Basic, 20% EMT-Intermediate, 13% EMT-Advanced and 8% EPs) completed the questionnaire. The mean age of participants was 32.7 ± 11.9 years. The years of experience in EMS ranged from 6 months to 45 years (mean 10 ± 8.8 years) with a monthly activity ranging from 4 to 220 hours (mean 75 ± 70 hours). 60% work as volunteers in the EMS.

### Participants´ opinions

75% of the participants already had experience with telemedicine, and 79% of them described tele-assistance as a very helpful tool. None of the participants with prior experience with telemedicine indicated that it was ‘rather not’ or ‘not useful’. As depicted in the table below, 88% of the participants stated that they could see themselves using telemedicine in prehospital EMS, and 71% reported that they had already been in situations in which telemedicine support would have been helpful (Table 1). Almost all healthcare professionals (95%) chose real-time data transmission as the preferred telemedicine modality for prehospital emergency situations.

**Table 1:** Telemedicine in prehospital EMS (single choice answer).

Could you envisage using telemedicine in prehospital EMS?	Percentages
Yes, definitely	48%
Yes, in certain cases	37%
No, rather not	5%
No, not at all	4%
Don’t know	3%
No response	3%

The subgroup analysis to search for associations between responses and respondent’s characteristic was done with an ordinary least squares linear regression model (S2 Table). The model fit was low at 0.218 r-squared value, likely due to the low number of participants with reservations against telemedicine and low number of subjects overall. In general, EMTs are more likely to favour telemedicine while Emergency Physicians are more critical (contained in the intercept). Acceptance was neither associated with sex or age. The subgroup analysis to test differences among different healthcare professions showed that all interviewed EMTs favour telemedicine while 50% of physicians are less accepting of the technology.

HCPs were also asked about concerns regarding telemedicine in EMS (Table 2). Technical complexity and time delays were the most common concerns. The majority (87%) would welcome tele-support for assistance with diagnostics determining the need for hospital admission (72%) and selecting the target hospital for patients (53%). 49% would prefer tele-support to be available only at the request of the EMT team on scene.

**Table 2:** Concerns about using telemedicine in the prehospital setting (multiple answers).

Do you have concerns about…?	Percentages
Technical complexity	41.4%
Time delays	29.3%
No concerns	27.3%
Personal restrictions	19.2%
Personal surveillance	16.2%
Data protection	15.2%
Other	11.1%

### Results of the qualitative data

The two focus groups comprised a total of 13 participants, representing a mix of full-time and volunteer EMTs and EPs. We derived 4 main codes and 19 subcodes in the thematic analysis (S1 Table). The results demonstrate that the expectations of telemedicine and the implementation of a telemedicine solution in EMS can be analysed on different, interrelated levels: the expectations related to the individual, organisational and technical level; and crucial external framework/regulatory conditions. In the following we present the key findings.

### Finding 1: Need for a specific mindset and the creation of a learning culture

EMTs and EPs (particularly younger ones) emphasise that the successful introduction and potential use of telemedicine within the organisation and among individual employees depends on a specific mindset. At the individual level, EMTs and EPs must have the necessary motivation and courage to implement a new process, especially if they have used other working methods for years or decades. At the organisational level, the step of asking for help and advice must be clearly encouraged. As two quotes indicate, efforts must be made to promote and foster a culture of mutual learning:

*“It needs to be normal in our corporate culture to request help. No matter whether you are an EMT or a physician–the person in need of support needs a contact point.” (Focus group 1)*

*"But mainly it is that I won’t be laughed at if I report that now*. *Is the issue big enough and important enough*, *and have I overlooked any aspect that I should have been able to judge myself*?*" (Focus group 1)*

Our data suggests that regular interdisciplinary exchanges in the form of training sessions and workshops would be particularly helpful as age and experience will have a strong impact on how EMTs handle situations. Those should include TEPs to enable learning across all professions in planned feedback loops. These sessions should also include structured training on the telemedicine tool itself. In addition, the implementation of a solution requires proactive communication with all stakeholders to allay fears and effectively integrate the solution into the existing organisational culture.

### Finding 2: Need to reduce in-person deployments of emergency physicians in the field

Our data shows that there is a high expectation that the TEP will make decisions, prescribe medications, and decide on admissions and discharges as EMTs are (often) not authorized to do so.

*“In my opinion, telemedicine should help avoid those missions where I don’t actually need an emergency physician with me next to the patient.” (Focus group 2)*

In rural areas, where EMTs regularly must wait up to half an hour for an EP to arrive, a telemedicine solution would have a tremendous impact on patient safety, as well as creating new scheduling and resource planning options. Another reason for involving TEPs during a deployment is legal protection. Especially in unclear cases, telemedicine could be used in a supportive way, since not every scenario can be packed into precise standard operating procedures and medical support could be very helpful here. However, the use of telemedicine should not cause EMTs to think less independently and rely solely on telemedicine for decision-making but should provide support when needed so that they feel confident in making decisions on site.

### Finding 3: Need for an understanding and experienced tele-emergency physician available via an easy-to-use telemedicine tool

According to our data, a telemedicine solution would be a viable option for about 10% but up to 30% of deployments. In such cases, it is essential that each step of the telemedicine process is conducted correctly. The TEP must radiate calm and be friendly, otherwise the solution will be used never again. The added value must be clear to all participants from the outset.

*“Someone with good personal skills who gives us the feeling of being in good hands, so that you feel well cared for and have no reservations in calling once too often rather than once too little.” (Focus group 1)*

In addition, the TEP should have experience in EMS, be able to communicate well, provide clear instructions, and have appropriate soft skills to offer adequate support. Additionally, the TEP should have further specialists directly at hand, such as a larger group or network of experts, or simply emergency physicians that are currently not on deployment. It is vital that telemedicine support is easily accessible, offering a direct line to a competent partner. With respect to technical expectations, our data demonstrates that simplicity and compatibility with existing solutions are deemed far more important than the technical details themselves. Focus group participants had many different ideas about telemedicine solutions, covering a wide range of scenarios: from a highly complex system such as a dedicated platform into which all data is fed and everything is available digitally, to a simple telephone connection that is considered sufficiently helpful in many situations.

## Discussion

Our findings demonstrate a clear need for telemedicine in EMS, with most HCPs and volunteer EMTs appreciating the option of decision support and treatment guidance. While the quantitative results provide a positive answer to the question of whether telemedicine is a viable option and identify possible application situations, the qualitative findings provide deeper insights into expectations and concerns at both the individual and organizational levels.

The data demonstrate that there is a strong desire for a learning culture, and a need to focus on cultural change within the organization to encourage people to ask for help. This desire was expressed mainly by participants with less professional experience. With this finding, it might be useful to let these”early adopters” convince those who have been in the system for a long time and do not feel such a strong need for the solution. Our finding that it is important to overcome potential fears and scepticism about the solution with a specific mindset is consistent with Beadry and Pinsonneault, who found that “fear of the solution” is one important factor that discourages HCPs from using technology and significantly reduces their willingness to use it [19]. Previous studies that evaluated digital transitions in emergency medicine recommend the implementation of a round table or platform for discussion in expert groups [20].

The timing for a telemedicine project seems very opportune as the willingness to access support in the form of a second opinion is becoming more widespread among HCPs, while COVID-19 has also enforced readiness to use technology [21]. In addition to the trend of technology use, another trend might have a positive impact on cultural change: with increasing specialisation within medicine and healthcare in general, it is becoming more and more difficult to work as a generalist. In each field, highly specialised professionals need to help one another, making it vital that they can cooperate closely [22].

Approximately 70% of the survey participants reported situations in which tele-support would have been helpful. EMTs’ demand for telemedicine solutions is understandable: in EMS healthcare providers face the challenge of limited information, time-critical emergencies, and environmental factors such as severe patient illness, noise levels and fatigue. Decisions and treatment strategies must be made in this complex environment [23]. Studies show that there is a desire for faster diagnosis; that it is not always necessary to have an EP on-site; and that remote diagnosis based on transmitting patient data supports accurate prehospital diagnosis [24]. Rossaint summarised a list of indications for circumstances in which input from TEPs would be appropriate. These include support in interpreting unclear electrocardiograms, refusal of transport, hypoglycaemia, and secondary transfers based on defined criteria [25]. Focus group participants estimated that telemedicine would be appropriate in up to 30% of all situations they encountered, and that they would need assistance primarily with diagnostics and selecting appropriate destination hospitals. Data from established telemedicine emergency systems regarding the proportion of tele-emergencies deployments in relation to the total number of deployments vary widely. It has been shown that consultation with a TEP can prevent unnecessary transports, although the extent of the reduction is unclear [26]. According to our data, next to the benefits for EMTs on an individual level, telemedicine could be very valuable in resource planning, given the wave of retirements and staff shortages.

Previous studies have found that HCPS’ intention to use new technologies depend on several factors, such as perceived usefulness and perceived ease of use [27]. Our data show few clear ideas about a potential solution, apart from the wish for ease of use. This reflects the literature on technology acceptance [28,29]. The ideas suggested by the focus group participants ranged from futuristic augmented reality to simple phone calls. There is evidence in the literature that the type of communication does not significantly determine its usefulness for diagnostic support [30]. Instead, physicians satisfaction and acceptance increase when patient data can be easily accessed [31].

Beside the expectation that the technology should not be too complex, study participants expressed ideas about the TEPs: experienced in EMS; kind and understanding; and able to explain and guide in a clear and structured manner. Interestingly, this is similar to the requirement profile defined by Felzen for their tele-EMS solution, with additional emphasis on emotional characteristics and soft skills [32].

Although our focus group participants viewed telemedicine positively, previous studies demonstrate that a major barrier for the acceptance of telemedicine solutions is the resistance from physicians (and other stakeholders) themselves [31]. This highlights the importance of a development and implementation process in which all stakeholders are involved right from the start, as stakeholders have specific experiences and knowledge to share, particularly in complex areas such as healthcare [12]. Thus, significant benefits can be realized when a truly community-based, collaborative development process is implemented. Here, a particular emphasis should be existing workflows when developing and integrating new solutions [20].

### Limitations

Participants voluntarily and actively participated, potentially attracting individuals with a technology affinity, interest in new solutions and a positive attitude towards telemedicine. With a sample size of 99 participants (response rate: 12.8%), the results of the survey offer a rather descriptive overview but must be cautiously analysed.

## Conclusion

This study indicates that telemedicine is currently a hot topic in EMS and receives strong support from EMTs. Expectations are that, in addition to technological solutions, attention is paid to a learning culture and proper mindset on an individual and organizational level as well as clarity through guidelines on when and how TEPs can be integrated to avoid in-person deployments.

## Supporting information

S1 File Questionnaire for “telemedicine in the rescue service—talk to us!” project. (DOCX)

S2 File Questions for the focus groups on the expectations and concerns on telemedicine in the emergency medical service. (DOCX)

S1 Table Codes of the thematic analysis of the focus groups. (DOCX)

S2 Table Regression model. (DOCX)
