# Supplementary material for: Expectations of healthcare professionals of community-based telemedicine in emergency medical service
Source: PLoS One. 2024 Sep 19;19(9):e0310895. doi: 10.1371/journal.pone.0310895 (PMC11412670; doi:10.1371/journal.pone.0310895)
Supplement: S1 File — (DOCX) [file pone.0310895.s001.docx]

**Telemedicine in the Rescue Service - Talk to us!**

**Telemedicine in the rescue service in Burgenland - an option?**

Welcome to the survey "Telemedicine in the rescue service in Burgenland - an option?

Due to technical progress, telemedicine is also increasingly used in preclinical medicine. But is the use of telemedicine really an option for the Burgenland rescue service? What potential, but also what challenges do the employees see in the implementation of telemedicine in the rescue service?

In the context of an Open Innovation in Science process, all active employees in the Burgenland rescue service are invited to share their experiences and opinions with us. These results will be discussed and interpreted in a group discussion and a workshop. Based on these results, concrete projects for the implementation of telemedicine in the Burgenland emergency medical services will be undertaken.

Answering the questionnaire will take about 5-7 minutes. We thank you very much for your commitment to help shape telemedicine projects in the rescue service through your participation in the survey.

**DATA PROTECTION**

Your data will be collected in anonymous form as part of the "Telemedicine in the Rescue Service" project. All information will be treated confidentially and in accordance with the German Data Protection Regulation (DSGVO) and will not be passed on to third parties.

**Field of activity:** (multiple selection possible)

O Emergency medical technician - Basic

O Emergency medical technician - Intermediate

O Emergency medical technician - Advanced

O Emergency physician
O Physician (General practitioners on-call service)

O Rescue control center employee

**Type of activity:** (multiple selection possible)

O Voluntary activity

O Professional activity

O Civilian service/Voluntary Social Year

**Where (district) do you work?** (multiple selection possible)

O Güssing

O Eisenstadt

O Mattersburg
O Neusiedl am See
O Oberpullendorf

O Oberwart

O Jennersdorf

O Not working in Burgenland

**How many years have you been working in the rescue service?**

_____ years

**How many hours do you work on average per month in the rescue service?**

_____ hours

**Do you have any experience with telemedicine consultation or support?**

O no

O yes

**If yes: In what form have you used this telemedical support?** (Multiple answer)

O Audio

O Video

O Data transmission (e.g. ECG, ultrasound, photos, ...)

**If yes: How helpful did you perceive this telemedical support to be?**

O very helpful

O rather helpful

O rather unhelpful

O not helpful

**Could you imagine using telemedicine in the rescue service (ambulance and rescue transport service)?**

O yes, in all cases

O yes, in certain cases

O no, rather not

O no, not at all

O don't know

**Do you have any concerns about telemedicine consultation or support in emergency services?** (Multiple selection possible)

O No concerns

O Concerns about supervision by superiors

O Concerns about patient privacy

O Concerns about personal limitations of the competence to act

O Concerns about technical complexity

O Concerns about time delays

O Other: _____

**Have you already had situations in the rescue service in which you would have liked telemedical support?**

O no

O yes: _____

**What telemedicine support in the ambulance service would you like to see?**(Multiple selection possible)

O Support in diagnostics (e.g. electrocardiogram)

O Support in therapeutic measures (e.g. medication administration)

O Support in the selection of the target hospital (e.g. stroke unit)

O Decision about leaving the patient on site

O Other: _____

O Don't know

**For which situations would you want telemedicine support?** (multiple selection possible)

O No support

O Acute illnesses (e.g. acute coronary syndrome, stroke, resuscitation)

O Chronic illnesses (e.g., leaving nursing patients)

O Other: _____

O Don't know

**What do you expect from telemedicine support in emergency medical services?**

(Multiple selection possible)

O Medical knowledge

O Decision-making competence

O Organizational competence

O Other: _____

O Don't know

**What level of competency would you expect from telemedicine support?**

(Multiple selection possible)

O Emergency medical technician with competencies

O Emergency physician

O Specialist (e.g. cardiology, neurology, pediatrics, ...)

O Other: _____

**Would you want telemedicine support only at the request of the rescue team?**

O yes, definitely

O yes, probably so

O no, probably not

O no, definitely not

O don't know

**Would you want telemedicine support on a routine basis as well (depending on appeal diagnosis)?**

O yes, definitely

O yes, probably yes

O no, probably not

O no, definitely not

O don't know

**What technical support would you expect to receive?**

O Audio

O Video

O Data transmission (e.g. ECG, ultrasound, photos, ...)

**Additional questions for physicians:**

**As a physician, could you imagine providing telemedical support in emergency medical services?**

O yes

O no

**If no: Why could you not imagine to provide telemedical support in emergency medical services?** (Multiple selection possible)

O Concept does not make sense

O Task not appealing

O Legal concerns

O Direct contact with patients is missing

O Other: _____

**If yes: For which professional group could you imagine offering telemedical support in the emergency medical services?** (Multiple selection possible)

O Emergency medical technician (all levels)

O Emergency physicians

O Physicians on call

O General practitioners (e.g. on home visits)

O Other: _____

**If yes: For which situations could you imagine offering telemedical support?**

O Consultation/transport decisions during interventions

O Taking medical history with patients via telephone/video chat

O Interpretation of point-of-care diagnostics

O Electrocardiogram

O Ultrasound

O Simple laboratory diagnostics

O Other: _____

**Finally, is there anything else you would like to share about telemedicine in emergency services?**

Free text:

**Gender:**

O female

O male

O diverse

**Year of birth:**

_____
