# Supplementary material for: Expectations of healthcare professionals of community-based telemedicine in emergency medical service
Source: PLoS One. 2024 Sep 19;19(9):e0310895. doi: 10.1371/journal.pone.0310895 (PMC11412670; doi:10.1371/journal.pone.0310895)
Supplement: S2 File — (DOCX) [file pone.0310895.s002.docx]

**Supplement 2**

**Questions for the focus groups on the expectations and concerns on telemedicine in the emergency medical service**

*Introduction*

*Indications for use*

- In what situation could you imagine using telemedicine support?
- What kind of support do you want?
- Which operations do you currently not have a good feeling about?
- How often would you currently like to have this support?
- In which questions would you like to have support?

*Training*

- What trainings would EMTs need to make this support useful? What content would be interesting? How should this content be taught?
- What training would physicians need for this support to be meaningful? What content? How should this content be taught?
- What other prerequisites are needed for the collaboration/training to work well? What else is needed beyond training?

*Infrastructure*

- What requirements do you have so that telemedicine can support you? Technical? Equipment? Personnel?

*General*

- What concerns and worries do you have when thinking about telemedicine support?
- What factors might be barriers to implementation?
- What potential do you see from telemedicine support?
