# Supplementary material for: Expectations of healthcare professionals of community-based telemedicine in emergency medical service
Source: PLoS One. 2024 Sep 19;19(9):e0310895. doi: 10.1371/journal.pone.0310895 (PMC11412670; doi:10.1371/journal.pone.0310895)
Supplement: S1 Table — (DOCX) [file pone.0310895.s003.docx]

**Supplement 3**

**Coding of the focus groups**

| ***Individual level*** | ***Organizational level*** | ***Technical level*** | ***Regulatory level*** |
| --- | --- | --- | --- |
| Education | Control center | Technical requirements | Law/ Regulations |
| Required skills | Resource plan / scheduling | Solution elements | Data protection and security |
| Decision support | Time factor | Implementation process | Medication |
| Personal connections / exchange | Learning culture |  | Patients / loved ones |
| Age / experience |  |  | Lack of staff |
| Situations |  |  | Regional differences |
