# Supplementary material for: Expectations of healthcare professionals of community-based telemedicine in emergency medical service
Source: PLoS One. 2024 Sep 19;19(9):e0310895. doi: 10.1371/journal.pone.0310895 (PMC11412670; doi:10.1371/journal.pone.0310895)
Supplement: S2 Table — (DOCX) [file pone.0310895.s004.docx]

**Supplement 4**

**Subgroup Analysis / Regression model**

Ordinary Least Squares linear regression model on Age, Sex and Medical Education to predict the acceptance of telemedicine support. Odds ratios are listed below.

|  | 5% | 95% | Odds Ratio |
| --- | --- | --- | --- |
| Intercept | 1.48 | 5.60 | 2.88 |
| Age | 0.46 | 2.43 | 1.06 |
| C(sex) [T.female] | 0.82 | 1.75 | 1.20 |
| C(Education) [T.EMT Basic] | 1.99 | 6.71 | 3.65 |
| C(Education) [T.EMT Intermediate] | 2.20 | 9.04 | 4.46 |
| C(Education) [T.EMT Advanced] | 2.66 | 9.55 | 5.04 |

Subgroup analysis of healthcare professionals

| **Could you envisage using telemedicine in prehospital EMS?** | | **Absolute number (percentage)** |
| --- | --- | --- |
| **EMT Basic (n=50)** | Yes, definitely | 25 (50%) |
|  | Yes, in certain cases | 21 (42%) |
|  | No, rather not | 3 (6%) |
|  | No, not at all | 1 (2%) |
| **EMT Intermediate (n=10)** | Yes, definitely | 6 (60%) |
|  | Yes, in certain cases | 4 (40%) |
| **EMT Advanced (n=20)** | Yes, definitely | 14 (70%) |
|  | Yes, in certain cases | 6 (30%) |
| **EP (n=6)** | Yes, in certain cases | 3 (50%) |
|  | No, rather not | 1 (17%) |
|  | No, not at all | 2 (33%) |
